# Supplementary material for: Molecular characterization of two recombinant isolates of telosma mosaic virus infecting Passiflora edulis from Fujian Province in China
Source: PeerJ. 2020 Feb 21;8:e8576. doi: 10.7717/peerj.8576 (PMC7039140; doi:10.7717/peerj.8576)
Supplement: Supplemental Information 1 [file peerj-08-8576-s001.pdf]

Table S1. Primers used for RT-PCR and Rapid Amplification of cDNA Ends (RACE) in this study

Primer set	Name	Sequence (5'-3')
S-1	S-1_P1	TNGCNATGACNCARTGGTGGA
	S-1_P2	CATTTTCAGCCAAWGGTCTT
S-2	S-2_P1	TGGTGGAATAGACAGYTGCA
	S-2_P2	CCCACATCATCCAGCCTCCA
S-3	S-3_P1	TCACAGTGCTCCAACAAGCC
	S-3_P2	TCTTTCCCTTGTATTGTGCC
S-4	S-4_P1	AAGTAAACTGAATAAGGAGG
	S-4_P2	TTTGTGATTTTCTGCAACCT
S-5	S-5_P1	GTTGTGGAGAATCAGTCTCA
	S-5_P2	ATTYACATCTCKTGCAGTGTGC
3′-RACE	3′-RACE_Outer Primer	TACCGTCGTTCCACTAGTGATTT
	3′-RACE_P1	GGTACAATGCCATCAAGATTGAGT
5′-RACE	5′-RACE_Long Primer	**C**TAATACGACTCACTATAGGGCAAGCAGTGGTATCAACGCAGAGT
	5′-RACE_Short Primer	CTAATACGACTCACTATAGGGC
	5′-RACE_P1	CAGTAGAACCAACCACCAGATGCC
	5′-RACE_P2	TGGATAAATGAAGCTCCCATCTTG
	5′-RACE_P3	CCACTATCACCTGGCTGGAT
